# A Case of Periodic Head-Ach, Effectually Removed by the Solutio Arsenica

**Published:** 1806-06

**Authors:** T. Weaver

**Affiliations:** Surgeon, of Walsall


A Case of Periodic Head-Ach, effectually removed by the Solutio Arsenica; *communicated by Mr.* T. Weaver, *Surgeon, of Walsall.*

THE present case is a further demonstration, and corroborating proof, of the beneficial effects resulting from the Solutio Mineralis, in addition to the many cases already communicated to the world, by Dr. Fowler and others, whose scientific knowledge shine conspicuous in the Annals of Medicine. The gentleman who is the subject of the present case, resides in this neighbourhood, and has been more than once afflicted with this singular and acute pain in the head, which, after the ineffectual administration of various internal remedies, usual upon such an occasion, the disease was removed by the application of leeches and blisters. From the favourable report of which, I was induced to adopt the same plan, but, unfortunately, not with equal success. The dolor capitis still continuing unabated, sometimes fixed near to the longitudinal sinus, at others, making a retreat towards the lambdoidal sutures, I thought it expedient to have recourse to other means, and considered it a favourable opportunity to try the effects of the mineral solution, so strongly recommended by Dr. Fowler; particularly as the paroxyms returned at regular and stated periods, usually commencing about eleven o'clock, A. M. and continuing without intermission until four P. M.

attended attended with nausea and vomiting. The disease was then suspended pro tempore, and he was consequently free from pain, until the usual time of its recurrence. The following draught was ordered, viz.

℞. Solut. arsenic. gtt. xv. Tinct. opii. gtt. x. Aq. pur. ℥iß. ft. haust. hor. 10ma. A. M. sumend. & rep. hor. 2d. P. M. & hora 6ta vespere.

The draughts were taken regularly, and occasioned, contrary to my expectations, no unpleasant sensations; the paroxysm returned the morning following, one hour before the usual time, but not so violent, and continued till two o'clock instead of four. Ordered the draughts to be repeated. The pain returned about eleven, and lasted till one, with less violence. As the stomach and bowels were not affected by the medicine, ordered the dose to be augmented ad guttas xviii.

3d. day, Repeated. A slight head-ach, not acute pain, and which continued but a short time.

4th day, Repeated the medicine, and no return of the paroxysm. Continued the medicine two days longer without any relapse, since which, he has been perfectly well, and free from any complaint.

From the above case, in conjunction with others, permit me to recommend the mineral solution, as an harmless and valuable medicine, worthy the notice and attention of medical practitioners, which I am certain, if used with prudence and discretion, can seldom, if ever, be productive of ill effects; but, like other valuable medicines, is liable to be abused, more particularly, if it perchance falls into the hands of empirics.

*April*, 1806.

